# Sinusoidal Endothelial Dysfunction Precedes Inflammation and Fibrosis in a Model of NAFLD

**DOI:** 10.1371/journal.pone.0032785

**Published:** 2012-04-03

**Authors:** Marcos Pasarín, Vincenzo La Mura, Jorge Gracia-Sancho, Héctor García-Calderó, Aina Rodríguez-Vilarrupla, Juan Carlos García-Pagán, Jaime Bosch, Juan G. Abraldes

**Affiliations:** Hepatic Hemodynamic Laboratory, Liver Unit, Hospital Clínic-IDIBAPS, CIBERrehd, University of Barcelona, Barcelona, Spain; University of Tor Vergata, Italy

## Abstract

Non-alcoholic fatty liver disease (NAFLD) is the hepatic manifestation of the metabolic syndrome. Most morbidity associated with the metabolic syndrome is related to vascular complications, in which endothelial dysfunction is a major pathogenic factor. However, whether NAFLD is associated with endothelial dysfunction within the hepatic vasculature is unknown. The aims of this study were to explore, in a model of diet-induced overweight that expresses most features of the metabolic syndrome, whether early NAFLD is associated with liver endothelial dysfunction. Wistar Kyoto rats were fed a cafeteria diet (CafD; 65% of fat, mostly saturated) or a control diet (CD) for 1 month. CafD rats developed features of the metabolic syndrome (overweight, arterial hypertension, hypertryglyceridemia, hyperglucemia and insulin resistance) and liver steatosis without inflammation or fibrosis. CafD rats had a significantly higher *in vivo* hepatic vascular resistance than CD. In liver perfusion livers from CafD rats had an increased portal perfusion pressure and decreased endothelium-dependent vasodilation. This was associated with a decreased Akt-dependent eNOS phosphorylation and NOS activity. In summary, we demonstrate in a rat model of the metabolic syndrome that shows features of NAFLD, that liver endothelial dysfunction occurs before the development of fibrosis or inflammation.

## Introduction

The metabolic syndrome is defined as a combination of abnormalities including central obesity, hypertriglyceridemia, low levels of HDL cholesterol, hypertension and hyperglycemia [1]. Insulin resistance (IR) is thought to be the pathophysiological hallmark of the syndrome [2], [3]. Non-alcoholic fatty liver disease (NAFLD) is the hepatic expression of the metabolic syndrome and has an increasing prevalence in the western population [4]. The spectrum of NAFLD lesions is wide, and goes from simple steatosis, non-alcoholic steatotohepatitis (inflammation, features of hepatocyte injury with or without fibrosis), to overt cirrhosis [5]. The mechanisms that account for disease progression in NAFLD are still poorly understood.

Most complications leading to morbidity in patients with the metabolic syndrome are of vascular origin [6]. One of the factors contributing to vascular disease in this setting is the presence of endothelial dysfunction, with decreased nitric oxide (NO) production [7], which has been consistently observed before cardiovascular events occur, and even before any pathological abnormalities in the vascular tree can be demonstrated [8]. This suggests that endothelial dysfunction is an early pathogenic event in the course of the vascular complications that occur in these patients. In keeping with this concept, correction of endothelial dysfunction is associated with an improvement in the rates of vascular events and, therefore, it is considered a useful therapeutic target in this syndrome [9], [10]. Interestingly, patients with NAFLD exhibit systemic endothelial dysfunction and a increased cardiovascular risk [11].

The liver sinusoidal endothelium is a very specialized and phenotypically differentiated endothelium, being its major specificities the presence of fenestrae and the absence of basal membrane [12]. Among other functions, an adequately functioning sinusoidal endothelium maintains an anti-inflammatory, anti-thrombotic and anti-fibrotic milieu within the liver parenchyma [13]–[15].

Some recent data have shown the presence of microvascular abnormalities in models of fatty liver, characterized by the presence of reduced sinusoidal perfusion [16] and structurally abnormal sinusoids due to lipid accumulation in parenchymal cells and to collagen deposition in the space of Disse [17]. However, the presence of liver endothelial dysfunction has not been specifically investigated. In addition, whether endothelial dysfunction might occur earlier than other features of advanced NAFLD (as it occurs in the peripheral circulation where endothelial dysfunction precedes the development of arteriosclerosis) is largely unknown.

The aims of this study were to characterize the changes in liver histology and liver microcirculatory function in a model of diet-induced obesity that expresses most features of the metabolic syndrome.

## Materials and Methods

### Animals, Diets, and Induction of Obesity

10 weeks old male Wistar Kyoto rats, weighing 225–250 grams, were caged individually in a 12∶12-hour light-dark cycle, temperature -and humidity-controlled environment. The rats were divided into two dietary conditions. The first group (CD; n = 15) was fed with standard chow diet for 1 month (which supplied 8% of calories as fat, type AO4; Panlab, Barcelona, Spain). The second group (CafD; n = 15) was fed with cafeteria diet for 1 month. Cafeteria diet is a highly palatable diet consisting of a daily offering of cookies, liver pate, bacon, standard chow, and whole milk supplemented with 333 g/litter of sucrose and 10 g/litter of a mineral and vitamin complex (Meritene; Nestle HealthCare Nutrition, Esplugues de Llobregat, Spain) as previously described [18], [19]. This diet contains 65% of the energy derived from fat that is predominantly saturated. All of the food items were weighed daily and presented in excess. Body weight was recorded daily. All rats were put on standard chow diet 24 hours before the experiments, to prevent any direct influence from the diet itself in the experimental results.

The animals were kept in environmentally controlled animal facilities at the Institut d’Investigacions Biomèdiques August Pi i Sunyer (IDIBAPS). All experiments were approved (ID: 3142, approved on 03/June/2005) by the Laboratory Animal Care and Use Committee of the University of Barcelona and were conducted in accordance with *Guide for the Care and Use of Laboratory Animals* (National Insitutes of Health, NIH Publication 86–23, revised 1996).

### Biochemical Measurements

Blood samples were taken in fasting conditions from the tail vein. Plasma was separated within 15 minutes and frozen at -80°C for subsequent analysis. Liver transaminases, glucose, bilirubin, triglycerides, free fatty acids (FFA) and insulin were analyzed with standard methods at the Hospital Clinic’s CORE lab.

To determine the liver triglycerides and FFA content 1g of frozen liver tissue was homogenized in 3mL of HEPES (1∶3 w/v) buffer composed of 50mM Tris, 150mM NaCl and 5mM EDTA (Sigma-Aldrich, Madrid, Spain) and analysed with standard methods at the Hospital Clinic’s CORE lab.

### Glucose Tolerance Test (GTT)

Rats were fasted for 4 hours before the administration of a glucose bolus (2 g/kg, i.p.; Braun Medical, Rubí, Spain). Glycemia was determined at 0, 15, 30, 60, 105 minutes after glucose administration with the AccuTrend glucose sensor (Roche Diagnostics, Sant Cugat del Valles, Spain).

### Intraperitoneal Insulin Sensitivity Test

Non-fasted rats were given an i.p. injection of insulin (1 U/kg; Actrapid, NovoNordisk, Novo Allé, Denmark), and blood glucose levels were measured at 0, 15, 30, 60, 120, and 180 minutes after injection.

### In vivo Hemodynamic Studies

Rats were anesthetized with ketamine (100 mg/kg, i.p., Imalgene, Barcelona, Spain) and midazolam (5 mg/kg, i.p., Laboratorio Reig Jofre, Barcelona, Spain) and maintained at constant temperature of 37±0.5°C (continuously monitored during the experiment). A tracheostomy and cannulation with a PE-240 catheter (Portex, Kent, UK) was performed in order to maintain adequate respiration during the anaesthesia. Thereafter, PE-50 catheters were introduced into the femoral artery and the ileocolic vein, in order to measure arterial pressure (MAP; mmHg) and portal pressure (PP, mmHg) respectively. A perivascular ultrasonic flow probe (2PR, 2-mm diameter. Transonic Systems Inc., Ithaca, NY, USA) placed around the portal vein measured the portal vein blood flow (PBF). Hepatic vascular resistance (HVR) was calculated as: PP*PBF^-1^. Results of flow and resistance were indexed to liver weight. All measurements were continuously registered on a multichannel computer based recorder (Powerlab 4SP, ADInstruments, Mountain View, LA).

### Isolated-Perfused Liver System

After haemodynamic measurements in vivo, livers were quickly isolated and perfused with Krebs’ buffer in a recirculation fashion with a total volume of 100 mL at a constant flow rate of 35 mL/min [20]. An ultrasonic transit-time flow probe (model T201; Transonic Systems, Ithaca, NY) and a pressure transducer were placed on line, immediately ahead of the portal inlet cannula, to continuously monitor portal flow and perfusion pressure. Another pressure transducer was placed immediately after the thoracic vena cava outlet for measurement of outflow pressure. The flow probe and the two pressure transducers were connected to a PowerLab (4SP) linked to a computer using the Chart version 5.0.1 for Windows software (ADInstruments, Mountain View, LA). The average portal flow, inflow and outflow pressures were continuously sampled, recorded and afterwards analyzed.

The perfused rat liver preparation was allowed to stabilize for 20 min before the studied substances were added. To assess the integrity of endothelial function, livers were preconstricted with metoxamine (Mtx) (10^−4^ M), an α-adrenergic agonist. After maximum vasoconstriction, increasing doses of the endothelium dependent vasodilator acetylcholine (ACh) (10^−7^, 10^−6^ and 10^−5^ M) were added. Another group of livers (CD n = 6; CafD n = 6) were perfused in the presence of the NO donor sodium nitroprusside (SNP; 10^−3^ M; Sigma-Aldrich, Madrid, Spain).

**Table 1:** Baseline characteristics and hemodynamics of rats fed for 1 month a control (CD) or a cafeteria (CafD) diet.

	Number of rats evaluated (CD/CafD)	CD	CafD	p
**Body weight (g): Baseline**	7/7	240±5	239±4	0.850
**Body weight (g): 4 weeks**	7/7	307±5	328±6	**0.001**
**Liver weight (g)**	7/7	9.1±0.3	11.1±0.4	**<0.001**
**% liver/total weight**	7/7	2.9±0.1	3.3±0.1	**0.005**
**Blood glucose (mg/dL)**	4/4	117±8	151±10	**0.003**
**Plasma insulin (µU/mL)**	4/4	35.9±4.5	56.9±8.4	**0.004**
**Bilirubin (mg/dl)**	4/4	0.10±0.04	0.28±0.10	**0.033**
**AST (U/L)**	4/4	85±14	88±8	0.656
**ALT (U/L)**	4/4	52±9	32±6	**0.014**
**Plasma FFA (µM)**	4/4	1613±274	3214±334	**0.001**
**Plasma Triglycerides (mg/dL)**	4/4	75±13	200±42	**0.026**
Liver FFA (umol*g liver-1)	4/4	9.2±1.1	13.0±1.1	0.070
Liver Triglycerides (mg*g of liver-1)	4/4	6.0±0.2	8.8±0.9	**0.035**
**Mean Arterial Pressure (mmHg)**	7/7	126.1±5	153.1±8	**0.005**
***In Vivo*** ** Portal Pressure (mmHg)**	7/7	7.5±2.1	9.7±0.6	0.062
Portal Blood Flow (mL*min-1*g of liver-1)	7/7	1.0±0.2	0.7±0.2	**0.050**
Hepatic Vascular Resistance (mmHg* g of liver*min* ml-1)	7/7	6.9±0.9	15.4±2.6	**0.027**

Data is presented as mean ± SD. (FFA: free fatty acids).

### Western Blot Analysis

After haemodynamic studies, liver samples were immediately frozen in liquid nitrogen and kept at −70 °C until processing. The samples were processed as previously described [21]. Briefly, aliquots from each sample containing equal amounts of protein (60–80 µg) were run on a SDS–polyacrylamide gel, and transferred to a nitrocellulose membrane. Equal loading was ensured by Ponceau staining. The blots were subsequently blocked for 1 h and probed overnight (at 4°C) with a mouse antibody recognizing eNOS (BD Transduction Laboratories, Lexington, KY), phosphorylated eNOS at Ser^1176^ (BD Transduction Laboratories, Lexington, KY), Akt (Cell Signaling Technology, Beverly, MA), P-Akt at Ser^473^, SE-1 (Santa Cruz Biotechnology, Santa Cruz, CA) or CD31 (Santa Cruz Biotechnology, California,CA ). This was followed by incubation with rabbit anti-mouse (1∶10,000) or goat anti-rabbit (1∶10,000) HRP-conjugated secondary antibodies (Stressgen, Glandford Ave, Victoria, BC, Canada) for 1 h at room temperature. Blots were revealed by chemiluminescence and digital images were taken by a luminescent image analyzer LAS-3000 (Fujifilm Life Science, Tokyo, Japan). Protein expression was determined by densitometric analysis using the Science Lab 2001, Image Gauge (Fuji Photo Film Gmbh, Düsseldorf). Quantitative densitometry values of proteins were normalized to β-Actin or GAPDH and displayed in histograms. The degree of eNOS phosphorylation at Ser^1176^ and Akt phosphorylation at Ser^473^ was calculated as the ratio between the densitometry readings of P-eNOS/eNOS and P-Akt/Akt bands.

**Figure 1:**
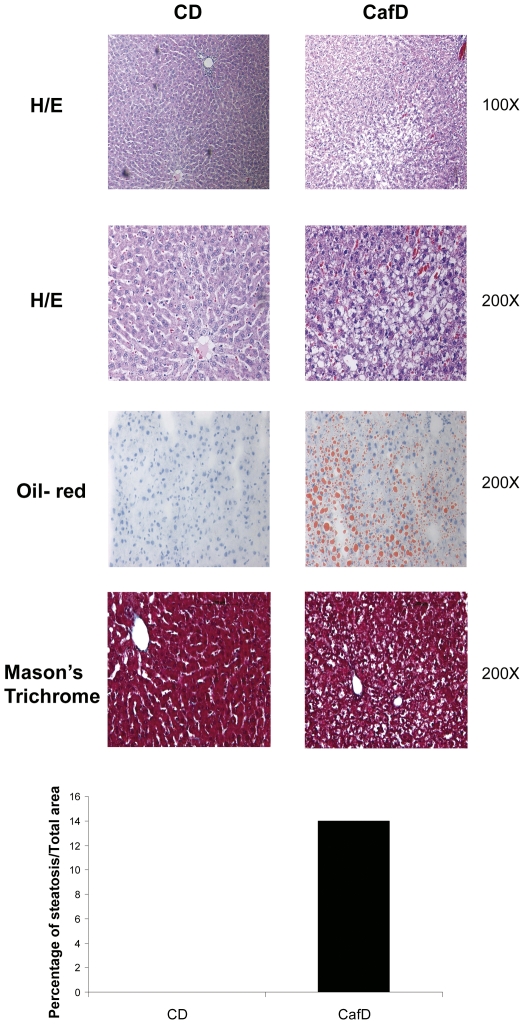
Hematoxylin/eosin (H/E), oil-red, Mason’s Thricrome images and steatosis area from livers from rats fed a control diet (CD) or a high fat diet (CafD). (original magnification, x100 or x200 as displayed in the figures).

To evaluate the effects of insulin on eNOS phosphorylation, rats (n = 12) from the two study groups were anesthetized with ketamine (80 mg/Kg) and midazolam (5 mg/Kg). Rats were injected with insulin (5 UI) or a similar volume (500 µL) of saline through the ileocolic vein [22]. Five minutes later the rats were euthanized and liver samples were obtained and immediately frozen in liquid nitrogen and kept at −70 °C until processing.

### Measurement of Nitric Oxide Synthase Activity

Nitric oxide synthase (NOS) activity was measured in homogenized livers from CafD and CD rats, by determining the conversion of 14C-labeled L-arginine to 14C-labeled L-citrulline, according to a previously reported method [23]–[25]. Enzymatic activity was expressed as nmol*min^-1^*mg^-1^protein.

**Figure 2:**
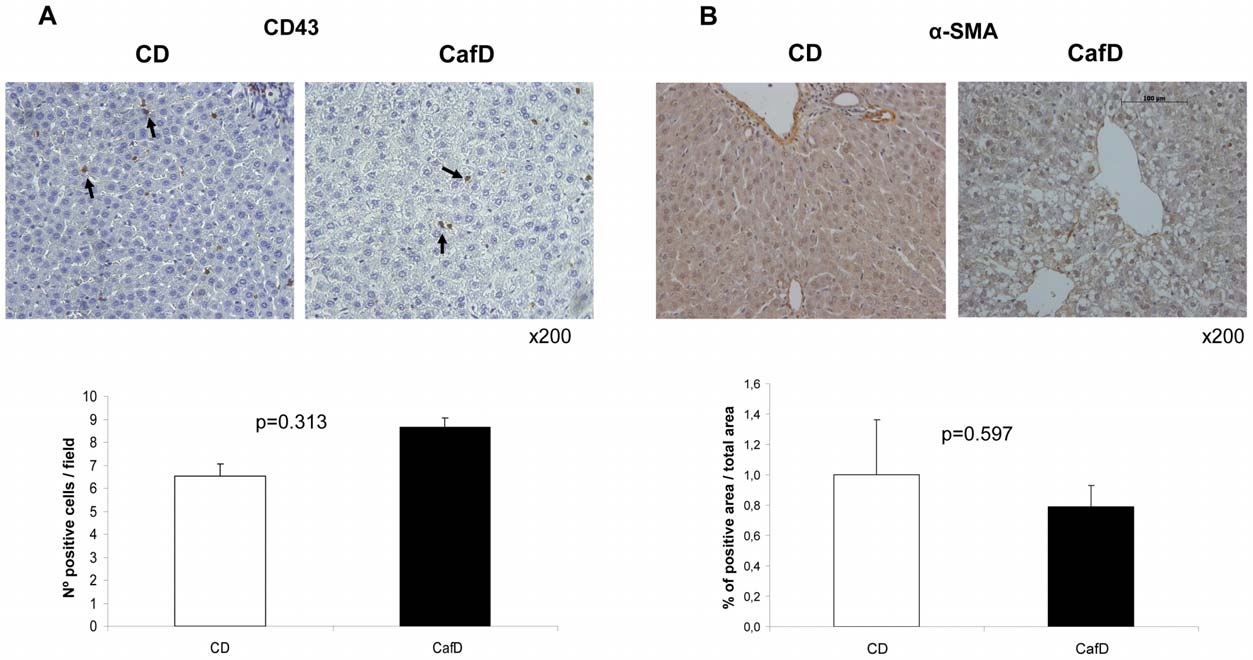
Liver inflammation and hepatic stellate cells phenotype. **A**) Immunohistochemistry showing CD43 (a pan-leukocyte marker) immunostaining in livers from high fat feeding (CafD) and control rats (CD). The administration for 1 month of a high fat diet did not induce liver inflammation (original magnification: x200). **B**) Cafeteria diet did not induce activation of HSC as assessed by immunohistochemistry for α-SMA.

### Histopathology

Liver samples were fixed in 10% formalin, embedded in paraffin and sectioned (thickness of 2 µm). Slides were stained with hematoxylin and eosin (H&E) and Mason’s Trichrome. Additional liver samples were frozen in liquid nitrogen, fixed in a freezing medium (Jung, Leica Microsystems, Nussloch, Germany) and stained with *Oil Red O* for 2 h at room temperature to detect neutral lipids. The samples were photographed, and analyzed using a microscope (Zeiss, Jena, Germany) equipped with a digital camera with the assistance of AxioVision softwares. The area of steatosis was quantified in 6 random photographs of each sample using AxioVision software.

**Figure 3:**
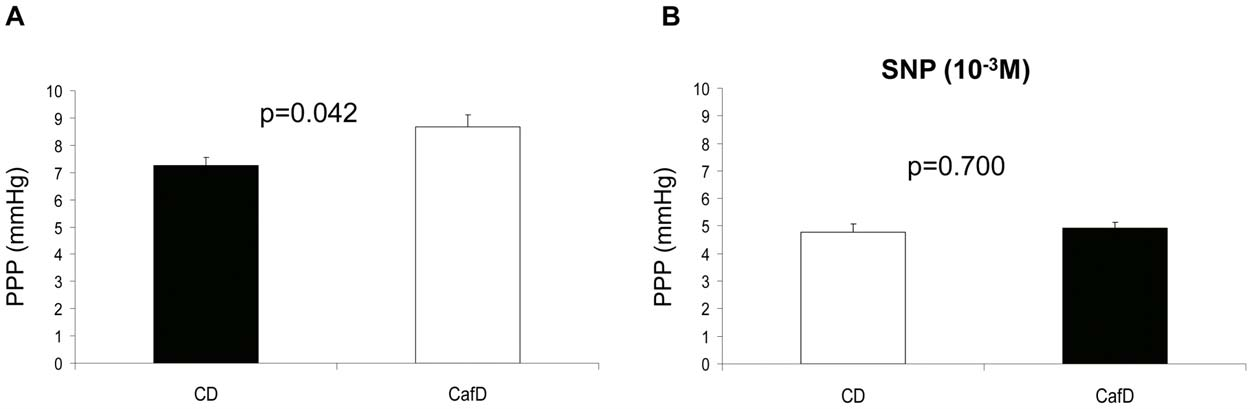
Ex-vivo assessment of liver circulation. Portal Perfusion Pressure (PPP) in CafD (n = 12) and CD rats (n = 12) in the absence (**A**) or the presence (**B**) of the NO donor sodium nitroprusside (SNP). Livers from rats fed cafeteria diet showed an increased PPP. No differences were observed in the presence of SNP (CD: control diet; CafD: cafeteria diet).

### Immunohistochemistry

Immunostaining of paraffin-embedded liver sections was performed with anti-CD43 (a pan-leucocyte marker) [26], anti-α-smotth muscle actin (α-SMA) or anti-CD34. Phosphate-buffered saline was used as a negative control. Bound antibody was visualized using diaminobenzidine as chromogen, and slides were then counterstained with hematoxylin. The number of CD43 positive cells was quantified using AxioVision software.

### Statistics

Statistical analysis was performed using the IBM SPSS 19.0 statistical package (IBM, Armonk, NY). Comparisons of the baseline characteristics between groups were performed with the unpaired Student’s *t*-test after confirming the assumptions of normality. Dose response curves were analysed with repeated measurements ANOVA introducing the type of diet as the between-subjects factor. All data are reported as means ± SD. Differences were considered significant at a *p* value <0.05.

**Figure 4:**
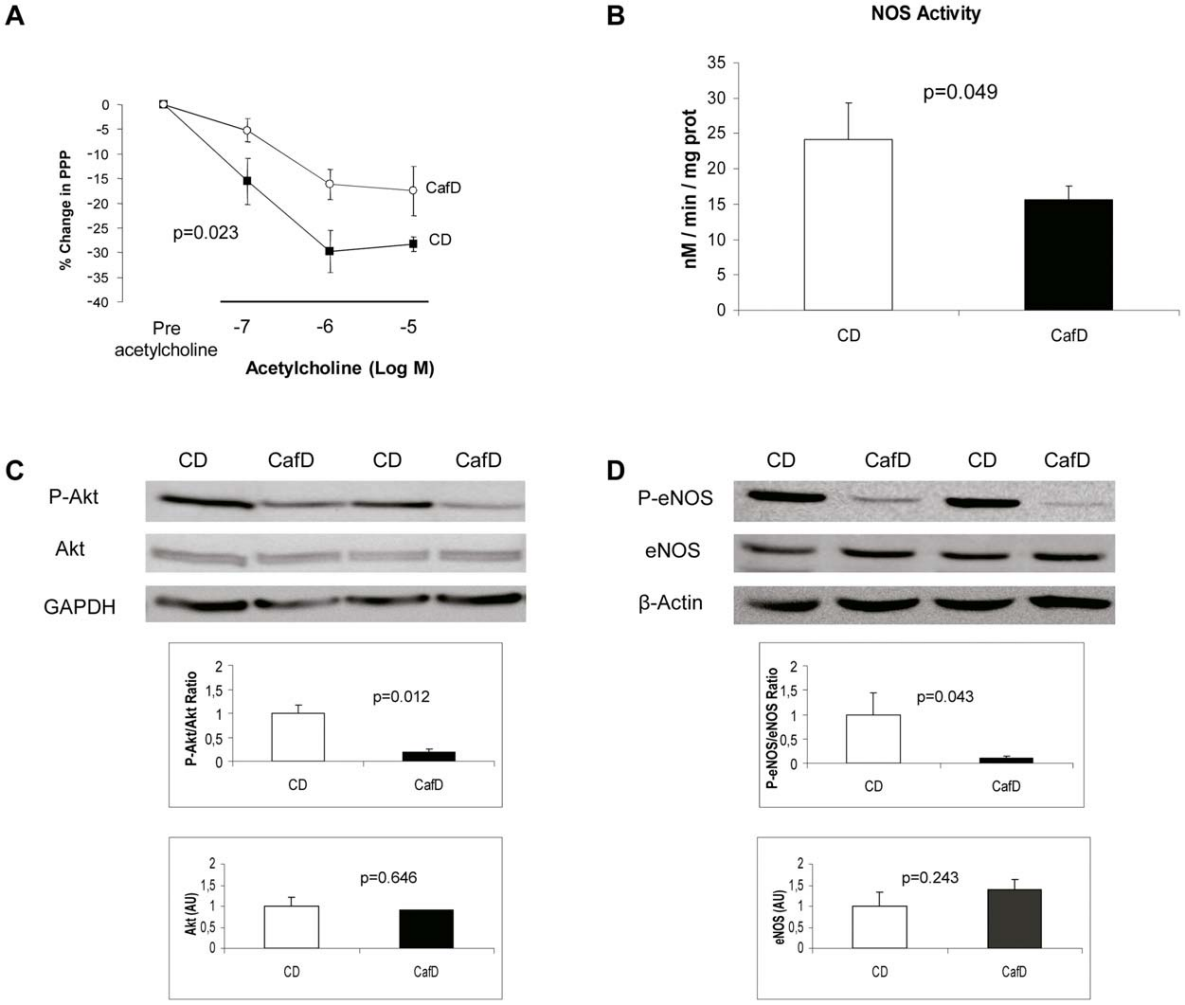
CafD rats show liver endothelial dysfunction. **A**) Response to ACh in livers from control diet rats (CD; black squares; n = 6) and high fat diet rats (CafD; white circles; n = 6). **B**) NOS activity in liver homogenates from control rats (CD; n = 4) and cafeteria fed-rats (CafD; n = 4). **C**) Representative blots and densitometry readings of liver P-Akt (at Ser^473^) to Akt ratio and **D**) P-eNOS (at Ser^1176^) to eNOS ratio (western blotting). AU: Arbitrary Units.

## Results

### One-month Cafeteria Diet Induces Features of the Metabolic Syndrome and NAFLD

Administration of high-fat palatable diet (cafeteria diet; CafD) for 30 days led to overweight, increasing the body weight gain by 33% as compared to rats fed the CD (p = 0.034). This was associated with other features of the metabolic syndrome: fasting hyperglycemia, a markedly abnormal glucose tolerance test, fasting hyperinsulinemia, impaired response to insulin (insulin resistance) and arterial hypertension (Figure S1 and Table 1). Plasma free fatty acids and triglycerides were markedly increased. (Table 1).

CafD increased liver weight, even after adjusting for body weight (p = 0.015) (Table 1). This was associated with an increase in liver triglyceride and FFA content. Bilirubin levels were slightly increased, AST was not changed and ALT was decreased. CafD rats showed marked liver steatosis (Fig. 1), mainly located at the pericentral areas, without inflammation (assessed with IHC for CD43) (Fig. 2A) or fibrosis (fig. 1). There was no increase in alpha smooth muscle actin expression (α-SMA), suggesting that at this early stage of NAFLD HSCs were not activated (Fig. 2B).

**Figure 5:**
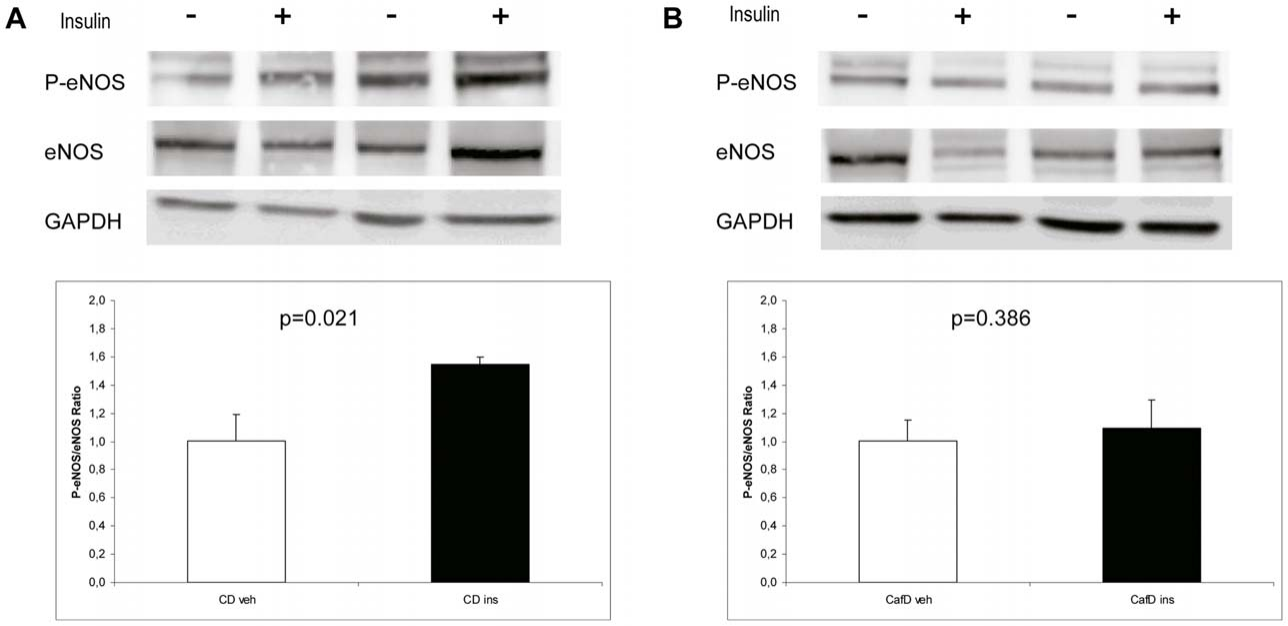
Representative blots and densitometry readings of liver P-eNOS (at Ser^1176^) to eNOS ratio (western blotting), five minutes after a portal injection of vehicle (white bars and -) or insulin (black bars and +). Insulin increased eNOS phosphorylation in CD rats (**A**) but not in CafD rats (**B**).

### Splanchnic and Systemic Hemodynamics after Cafeteria Diet

CafD induced arterial hypertension and a slight, but not significant, increase in *in vivo* portal pressure (Table 1). This was associated with a decrease in portal blood flow, indicating that CafD significantly increased *in vivo* intrahepatic vascular resistance (Table 1).

### CafD induces Endothelial Dysfunction at the Liver Microvasculature

To further characterize the abnormalities of the intrahepatic microcirculation induced by CafD we conducted experiments in the isolated perfused liver. *Ex- vivo* liver perfusion evaluates the liver microcirculation devoid of extra-hepatic influences, such as vasoactive mediators or changes in portal blood inflow, since the liver is perfused with a *clean* buffer at a constant flow. The *ex-vivo* portal perfusion pressure (PPP) was significantly increased in CafD rats as compared to CD rats (Fig. 3A). This difference disappeared when livers were perfused in the presence of the NO donor SNP (Fig. 3B), suggesting that the increase in PPP was due to an increased hepatic vascular tone.

One of the canonic features of endothelial dysfunction is a decreased response to the endothelium dependent vasodilator acetylcholine (ACh). Therefore, after pre-constriction with methoxamine, we tested the response of the liver vasculature to increasing doses of ACh. The vasodilating response to ACh was significantly blunted in livers from CafD rats as compared to CD rats, thus showing that one-month cafeteria diet induces sinusoidal endothelial dysfunction (Fig. 4A).

**Figure 6:**
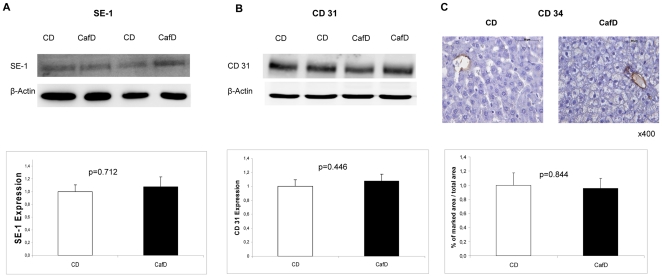
Endothelial cells phenotype. Cafeteria diet did not induce changes in **A**) SE-1 expression nor **B**) CD31, which are closely correlated with fenestrations and capillarization at the sinusoidal endothelial cells. **C**) Cafeteria diet did not induce *de novo* expression of CD34, a marker of loss of LSEC phenotype.

A well characterized mechanism that leads to systemic endothelial dysfunction in the metabolic syndrome is a decreased Akt-dependent eNOS phosphorylation (at Ser^1176^) at the vascular endothelium, with ensuing decreased eNOS activity. To evaluate whether this was also the case at the intrahepatic circulation in our NAFLD model, we assessed Akt and eNOS phosphorylation in liver samples from CafD and CD rats. Hepatic eNOS is majorly expressed at the liver endothelium, and therefore it can be safely assumed that changes in eNOS phosphorylation in total liver are representative of changes at the endothelium (Figure S2). Both P-Akt (at Ser^473^, the active form of Akt) and P-eNOS were significantly decreased in CafD (Fig. 4C and 4D). Furthermore, liver NOS activity was decreased in CafD as compared to CD fed rats (Fig. 4B). Altogether, these results reinforce the concept that the liver endothelium exhibits endothelial dysfunction in our experimental model of early NAFLD.

To evaluate whether CafD impairs insulin to eNOS signalling at the liver endothelium we assessed the degree of eNOS phosphorylation (at Ser^1176^) after a portal injection of insulin (5UI) or saline. Insulin increased P-eNOS/eNOS ratio in CD rats but not in CafD rats (Fig. 5), indicating that CafD induces liver endothelial insulin resistance.

The liver sinusoidal endothelium has differential characteristics from the vascular endothelium, among others the presence of fenestrae. One of the changes associated with advanced liver disease is a phenotypical shift in LSECs, including a loss in fenestrations. In vitro data suggest that the degree of LSEC fenestrations is closely reflected by the expression of SE-1 [27]. CafD did not decrease SE-1 expression, indirectly suggesting intact fenestrae in our model (Fig. 6A). An additional feature that reflects the loss of the typical LSEC phenotype is the *de novo* expression of CD31 and CD34 at the sinusoids [28]–[33]. Both are expressed by vascular endothelial cells, but not LSECs [34]. There were no changes in liver CD31 expression between the two groups (Fig. 6B). In addition, IHC did not show *de novo* expression of CD34 at the sinusoids after one-month CafD (Fig. 6C). Altogether, therefore, our data suggest that liver endothelial dysfunction occurs in our model of NAFLD earlier than inflammation, fibrosis or morphological changes in LSEC.

## Discussion

Endothelial dysfunction is a major factor implicated in the development of arteriosclerosis and vascular complications in patients with the metabolic syndrome [35], [36]. However, the endothelial phenotype and the regulation of endothelial function might have significant dissimilarities among different organs and tissues. Even within a single vascular territory there might be major differences in endothelial function between macro and microcirculations. Therefore, the pathophysiological events present in the peripheral vasculature cannot be directly extrapolated to the liver vasculature.

The integrity of the liver sinusoidal endothelium is of utmost relevance for the maintenance of liver physiology, and disruption of sinusoidal endothelial function might have a prominent role in liver pathophysiology. Liver sinusoidal endothelial dysfunction, with decreased intrahepatic NO production, has been considered for years a relevant pathogenic factor in the progression of liver cirrhosis [37]. It has been demonstrated that decreased NO production contributes to increased intrahepatic vascular resistance and, therefore, to portal hypertension, but it is also though to contribute to other relevant mechanisms implicated in disease progression. An adequately functional sinusoidal endothelial cell tonically inhibits, through NO production, the activation of hepatic stellate cells (HSC) [14], [38], thus being a potent natural antifibrotic. In addition, endothelial derived NO protects from microthrombotic events within the sinusoids [15], a well described mechanism of cirrhosis progression [39], [40]. Further, a healthy sinusoidal endothelium is essential for liver regeneration [34]. Though intrahepatic endothelial dysfunction is most severe in advanced phases of cirrhosis (with ascites), less advanced disease (established cirrhosis without ascites) is still associated with (milder) endothelial dysfunction [41]. However, it is unknown whether sinusoidal endothelial dysfunction might precede the development of fibrosis at the liver circulation., as it occurs in peripheral vascular disease in which endothelial dysfunction occurs earlier than structural changes of arteriosclerosis and is believed to represent the initial pathogenic event [42], [43]. This could have a major therapeutic relevance, since liver sinusoidal endothelial dysfunction constitutes a druggable target with compounds already available in the market, such as statins [21], [44].

In the present study we show, for the first time, functional features of intrahepatic endothelial dysfunction in a model of early NAFLD. We first show, in the complex *in vivo* setting, an increased hepatic vascular resistance (calculated from directly measured portal blood flow and portal pressure). To more precisely assess the abnormalities in the liver microcirculation we performed further studies in the isolated and perfused liver, demonstrating an impaired vadodilatory response of the liver vascular bed to acetyl-choline, the hallmark feature of endothelial dysfunction. In addition, we provide evidence of impaired endothelial function at the molecular level, showing decreased Akt-dependent eNOS phosphorylation. We and others [45] have demonstrated that liver eNOS expression is negligible outside endothelial cells, and so it can be safely assumed that changes in eNOS phosphorylation in liver homogenates represent changes at the liver endothelium. Specific phenotypical markers of LSECs, such as absence of CD34 and CD31 expression, were maintained in livers from CafD group. In addition, there were no changes in SE-1 expression, which has been shown (in *in-vitro* experiments) to closely reflect the degree of fenestrations. Thus, we provide here functional and molecular evidence of a dysfunctioning liver sinusoidal endothelium in the presence of phenotypically normal LSECs.

A major mechanisms that has been implicated in the development of endothelial dysfunction in the metabolic syndrome at peripheral vessels is insulin resistance itself, since insulin, via Akt [46], stimulates endothelial NO release through a Ca^2+^ independent pathway. Disruption of insulin signalling specifically at the endothelium impairs endothelial dependent vasodilation [47], [48]. We have recently shown that insulin signalling at the liver endothelium is disrupted as early as after 3 days of a high fat administration [49], and we confirm here this finding in our CafD model, suggesting that this mechanism could probably be a major contributor to the development of liver endothelial dysfunction in NAFLD.

Our model was appropriate to test our hypothesis, since it reproduces most features of the metabolic syndrome, such as overweight, arterial hypertension, hypertrigliceridemia and insulin resistance, and that causes liver steatosis not associated with inflammation or fibrosis, thus mimicking early human NAFLD. This model, based in the administration of a high fat diet with a lipid content that resembles that of “fast food”, has been thoroughly used in metabolic studies, but data concerning the liver abnormalities associated with “cafeteria diet” are scant. The high content of saturated fat in this model, in contrast to high fat diets with high polyunsaturated fat content, that are associated with markedly increased levels of oxidative stress and which rapidly induce inflammation [50], probably reflects better the type of unhealthy diets that lead to NAFLD and the metabolic syndrome in clinical practice. In addition, this model could compare favourably as a model of NAFLD with genetic models of obesity such as *fa/fa* rats or *ob/ob* mice, in which critical pathways involved in liver injury are inactive, or with other widely used models such as methionine-choline deficient diet, which does not exhibit insulin resistance [51].

In summary, in this study we demonstrate, in a rat model of the metabolic syndrome that shows features of NAFLD, that liver endothelial dysfunction occurs before the development of fibrosis or inflammation. Therefore, liver endothelial dysfunction might be an early event implicated in disease progression in NAFLD, and might constitute a useful target for devising therapies for this disease.

## Supporting Information

Figure S1 **Rats fed a high fat diet (black squares) developed overweight and an impaired response to the glucose tolerance and insulin sensitivity tests.** A) Body weight in CD and CafD during the study period. B) Glucose tolerance test. Blood glucose levels after an intraperitoneal (i.p.) injection of glucose (2 g/Kg) (n = 4 rats per group). C) Insulin sensitivity test. Glucose levels after an i.p. of insulin (5UI) (n = 4 rats per group).
(TIF)

Figure S2 **Protein expression of eNOS and Akt in the different liver cell types. GAPDH was used as a loading control:** eNOS is detected selectively in LSECs. **(LSEC: liver sinusoidal endothelial cells. HSC: Hepatic stellate cells).** Methods: LSEC, Kuppfer cells and hepatocytes were isolated from CD rat livers (n = 3) as described previously *(Gracia-Sancho J. et al. Hepatology 47: 1248–1256)*. Briefly, after perfusion of the livers with collagenase, hepatocytes and non-parenchymal cells were separated by centrifugation. Hepatocytes were washed twice with PBS and immediately lysed. Kuppfer cells and LSEC were isolated by isopycnic sedimentation (through a two-step density gradient Percoll) and pure monolayers were established by selective attachment on plastic or on collagen I, respectively. Cells were lysed after 12h of culture. Hepatic stellate cells were isolated from CD livers (n = 3) as described previously *(Rodriguez-Vilarrupla A, et al. Liver Int 28: 566–573)*. Briefly, livers were perfused with Gey’s balanced salt solution (GBSS), and digested at 37 °C with 0.01% collagenase, 0.01% DNAse and 0.004% pronase in GBSS. Cells were centrifuged at 50*g*, the supernatant was centrifuged at 800*g* and the pellet was then washed two times with Roswell Park Memorial Institute medium. Cells were grown in Iscove’s modified Dulbecco’s medium and lysed 3–5 days after isolation. Hepatic primary cells were homogenized in triton-lysis buffer. Aliquots from each sample containing equal amounts of protein (20 µg) were run on an 8% sodium dodecyl sulfate–polyacrylamide gel and transferred to a nitrocellulose membrane.
(TIF)
