# Supplementary figures and images for: Sinusoidal Endothelial Dysfunction Precedes Inflammation and Fibrosis in a Model of NAFLD
Source: PLoS One. 2012 Apr 3;7(4):e32785. doi: 10.1371/journal.pone.0032785 (PMC3317918; doi:10.1371/journal.pone.0032785)

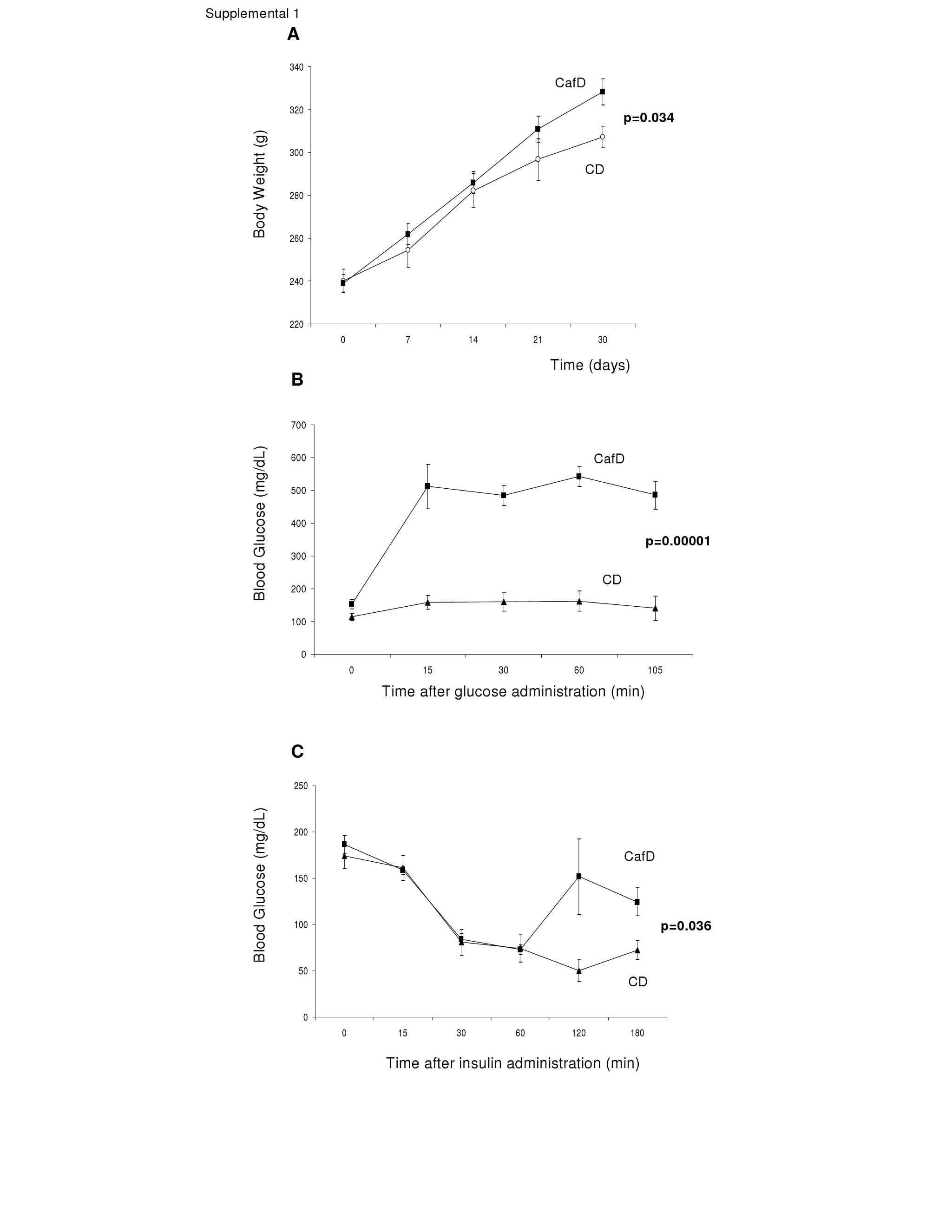

Supplement: Figure S1 — Rats fed a high fat diet (black squares) developed overweight and an impaired response to the glucose tolerance and insulin sensitivity tests. A) Body weight in CD and CafD during the study period. B) Glucose tolerance test. Blood glucose levels after an intraperitoneal (i.p.) injection of glucose (2 g/Kg) (n = 4 rats per group). C) Insulin sensitivity test. Glucose levels after an i.p. of insulin (5UI) (n = 4 rats per group). (TIF) [file pone.0032785.s001.tif]

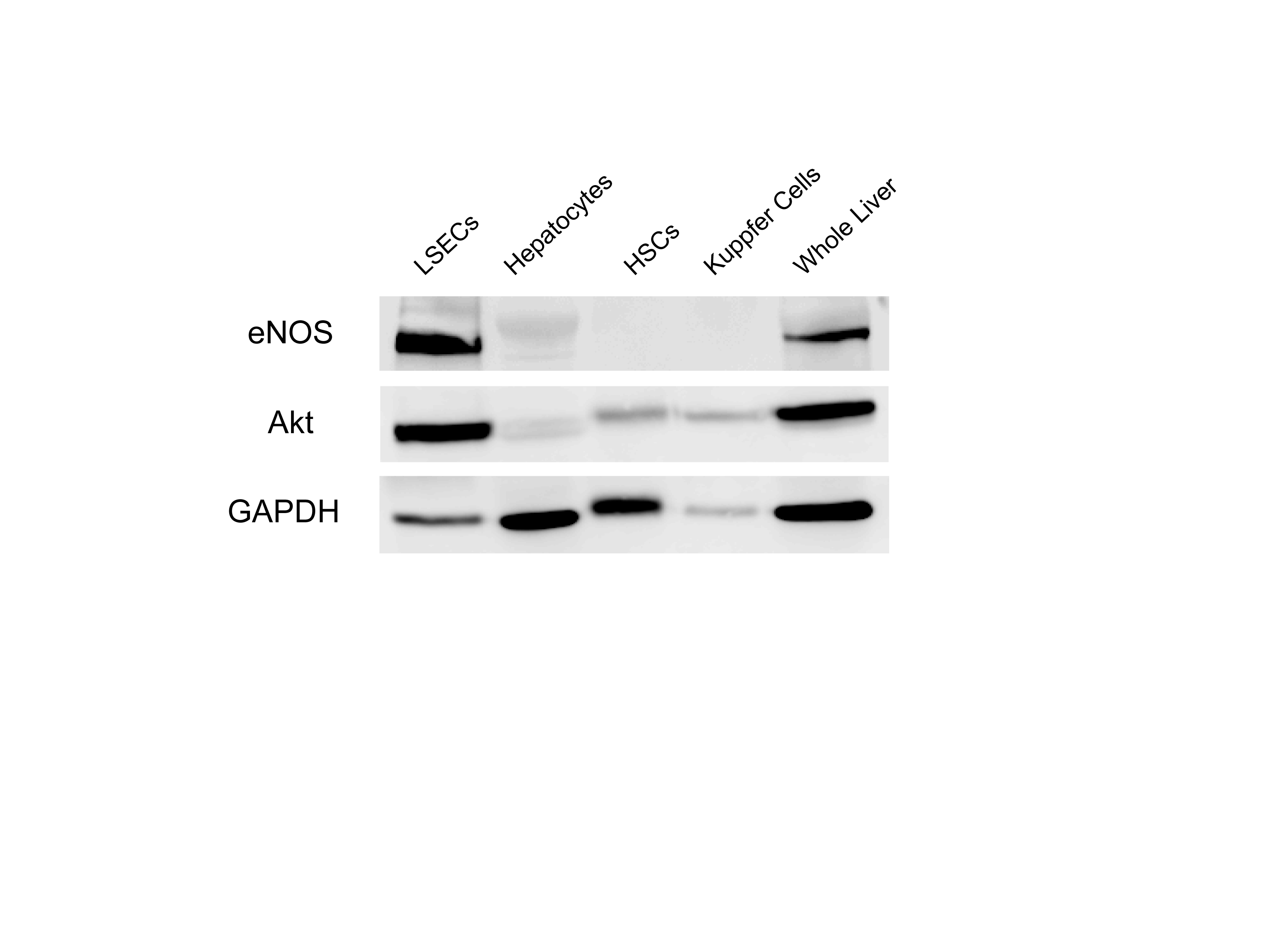

Supplement: Figure S2 — Protein expression of eNOS and Akt in the different liver cell types. GAPDH was used as a loading control: eNOS is detected selectively in LSECs. (LSEC: liver sinusoidal endothelial cells. HSC: Hepatic stellate cells). Methods: LSEC, Kuppfer cells and hepatocytes were isolated from CD rat livers (n = 3) as described previously (Gracia-Sancho J. et al. Hepatology 47: 1248–1256). Briefly, after perfusion of the livers with collagenase, hepatocytes and non-parenchymal cells were separated by centrifugation. Hepatocytes were washed twice with PBS and immediately lysed. Kuppfer cells and LSEC were isolated by isopycnic sedimentation (through a two-step density gradient Percoll) and pure monolayers were established by selective attachment on plastic or on collagen I, respectively. Cells were lysed after 12h of culture. Hepatic stellate cells were isolated from CD livers (n = 3) as described previously (Rodriguez-Vilarrupla A, et al. Liver Int 28: 566–573). Briefly, livers were perfused with Gey’s balanced salt solution (GBSS), and digested at 37 °C with 0.01% collagenase, 0.01% DNAse and 0.004% pronase in GBSS. Cells were centrifuged at 50g, the supernatant was centrifuged at 800g and the pellet was then washed two times with Roswell Park Memorial Institute medium. Cells were grown in Iscove’s modified Dulbecco’s medium and lysed 3–5 days after isolation. Hepatic primary cells were homogenized in triton-lysis buffer. Aliquots from each sample containing equal amounts of protein (20 µg) were run on an 8% sodium dodecyl sulfate–polyacrylamide gel and transferred to a nitrocellulose membrane. (TIF) [file pone.0032785.s002.tif]
